# MVST: Identifying spatial domains of spatial transcriptomes from multiple views using multi-view graph convolutional networks

**DOI:** 10.1371/journal.pcbi.1012409

**Published:** 2024-09-05

**Authors:** Hao Duan, Qingchen Zhang, Feifei Cui, Quan Zou, Zilong Zhang

**Affiliations:** 1 School of Computer Science and Technology, Hainan University, Haikou, China; 2 Institute of Fundamental and Frontier Sciences, University of Electronic Science and Technology of China, Chengdu, China; 3 Yangtze Delta Region Institute (Quzhou), University of Electronic Science and Technology of China, Quzhou, China; Johns Hopkins University Whiting School of Engineering, UNITED STATES OF AMERICA

## Abstract

Spatial transcriptome technology can parse transcriptomic data at the spatial level to detect high-throughput gene expression and preserve information regarding the spatial structure of tissues. Identifying spatial domains, that is identifying regions with similarities in gene expression and histology, is the most basic and critical aspect of spatial transcriptome data analysis. Most current methods identify spatial domains only through a single view, which may obscure certain important information and thus fail to make full use of the information embedded in spatial transcriptome data. Therefore, we propose an unsupervised clustering framework based on multiview graph convolutional networks (MVST) to achieve accurate spatial domain recognition by the learning graph embedding features of neighborhood graphs constructed from gene expression information, spatial location information, and histopathological image information through multiview graph convolutional networks. By exploring spatial transcriptomes from multiple views, MVST enables data from all parts of the spatial transcriptome to be comprehensively and fully utilized to obtain more accurate spatial expression patterns. We verified the effectiveness of MVST on real spatial transcriptome datasets, the robustness of MVST on some simulated datasets, and the reasonableness of the framework structure of MVST in ablation experiments, and from the experimental results, it is clear that MVST can achieve a more accurate spatial domain identification compared with the current more advanced methods. In conclusion, MVST is a powerful tool for spatial transcriptome research with improved spatial domain recognition.

## Author summary

Spatial transcriptome sequencing can not only reveal the mechanisms of disease development, but can also be used to explore the structure of biological tissues, which are widely used in a variety of fields such as developmental biology and oncology. To utilize spatial transcriptome data to understand how different cells work together to perform complex functions, spatially relevant groups of cells must be identified, which leads to the task of dissecting tissues into spatial domains. However, most of the currently available spatial domain identification tools have yet to fully utilize the information embedded in spatial transcriptomic data, and their identification remains to be improved for the fine-grained exploration of tissue structure. Here, we developed a tool for accurate spatial domain identification, and our tool makes full use of the various types of spatial transcriptome data from multiple perspectives, identifying the real grouping information in spatial transcriptome data as much as possible. Our results show that our spatial domain identification tool can identify spatial domains more accurately and provide effective help for biological organization structure exploration.

## Introduction

To explore the spatial structure of tissues and the spatial expression patterns of genes, numerous spatial transcriptome (ST) sequencing technologies have been developed to quantify the expression of a large number of genes in the spatial context of tissues and cells [1–3]. Transcript information obtained using spatial transcriptome technologies can correspond to tissue locations, enabling the restoration of gene expression status at various spatial locations within a tissue [4–6]. From the development history of spatial transcriptome sequencing technologies, they can be meticulously classified into five subcategories at present: i) technologies based on microdissected gene expression, e.g., LCM [7], ProximID [8]; ii) in situ hybridisation (ISH) technologies, e.g., seqFISH [9,10], MERFISH [11], osmFISH [12]; iii) in situ sequencing (ISS) technologies, such as STARmap [13]; iv) in situ capturing technologies, such as Slide-Seq [14], HDST [15], and the current mainstream spatial transcriptome sequencing technology-10X Visium (https://www.10xgenomics. com/), and v) in silico reconstruction of spatial data [16]. Spatial transcriptomics can not only help elucidate the spatial dynamics of normal organ development but also assist in the study of the mechanisms of disease onset and progression [17], and play a significant role in exploring the tumor microenvironment [18–21].

Identifying spatial domains refers to identifying regions with similarities in gene expression and histology. Identifying spatial domains is the most basic and critical aspect of spatial transcriptome data analysis, and is one of the major challenges in spatial transcriptome research. The identification of spatial domains can further demonstrate the spatial dependence of gene expression and provide a basis for further studies such as spatially variable gene identification. The identification of spatial domains can be achieved using traditional clustering methods and analytical tools developed for spatial domain identification.

Earlier methods used for spatial domain recognition, that is, traditional clustering methods such as k-means [22,23] and Louvain[24], which do not consider spatial location, are not ideal. Various spatial domain recognition methods have been developed that combine spatial location information, gene expression data, and histopathological images to better identify the spatial domains. For example, BayesSpace [25] achieves spatial domain recognition based on a spatial prior, that is, neighboring points have a high probability of belonging to the same cluster. Giotto [26] used a Hidden Markov Random Field model and spatial neighbors prior to identifying spatial domains. Currently, rapidly developing deep learning models such as graph neural networks have been applied to spatial domain recognition with good results. For example, SEDR [27] uses a variational graph self-encoder to embed spatial information into low-dimensional representations of gene expression to identify spatial domains. SpaGCN [28] identifies spatial structural domains by aggregating gene expressions from neighboring points using graph convolutional neural networks. CCST [29] employs an unsupervised graph contrast learning model with mutual information maximization to learn node embeddings for identifying spatial domains. STAGATE [30] used a graph attention self-encoder to aggregate spatial and gene expression information to identify spatial domains and employed an attention mechanism to better explore spatial similarities at the boundaries of spatial domains. STMGCN [31] uses a graph convolutional network-based encoder to learn specific representations of multiple topological graphs to identify spatial domains. CONGI [32] uses contrast learning to adapt gene representations to image information and to accurately identify spatial domains. Most of the above methods construct adjacency matrices based on spatial locations and do not make full use of information, such as gene expression data and histopathological images, to construct graph structures. Currently, studies have shown that transcriptomics data and histopathology images have a common link [33], and more studies have achieved better results in spatial transcriptomics prediction, exploration of molecular features of spatial transcriptomes, prediction of clinical outcomes, and noise correction of spatial transcriptomics data by using histopathology image information [34–36], Therefore, increasing the analysis of histopathology images can provide more comprehensive information for spatial transcriptomics-related studies, including spatial domain identification, to some extent. Although some methods also consider the use of information such as histopathological images, they do not analyze them in an all-round and multi-perspective manner, which may result in missing important information characterizing spatial expression patterns.

In this study, we propose a spatial domain recognition method, MVST, based on a multi-view graph convolutional network, which can combine gene expression data, spatial location information, and histopathological image information to learn low-dimensional embedding and achieve accurate recognition of spatial domains. MVST first constructs three undirected graphs based on gene expression data, spatial location, and histopathological images to represent the dependency of the data and features obtained by the dimensionality reduction of gene expression data as attributes of graph nodes. Second, low-dimensional graph embeddings are learned from each undirected graph by a multiview graph convolutional encoder with an attention mechanism and further fed into a coherent embedding encoder to obtain consistent cluster embeddings among multiple views, which are then used for spatial domain recognition. We tested the clustering performance of MVST using three real datasets from the current mainstream platform for spatial transcriptomes, 10x Visium, and some simulated datasets constructed based on the real datasets, as well as ablation experiments, and the results of the experiments show that compared with the current more advanced methods MVST has certain advantages in clustering analysis and can identify spatial domains more accurately.

## Results

### Overview of the MVST

**Fig 1:**
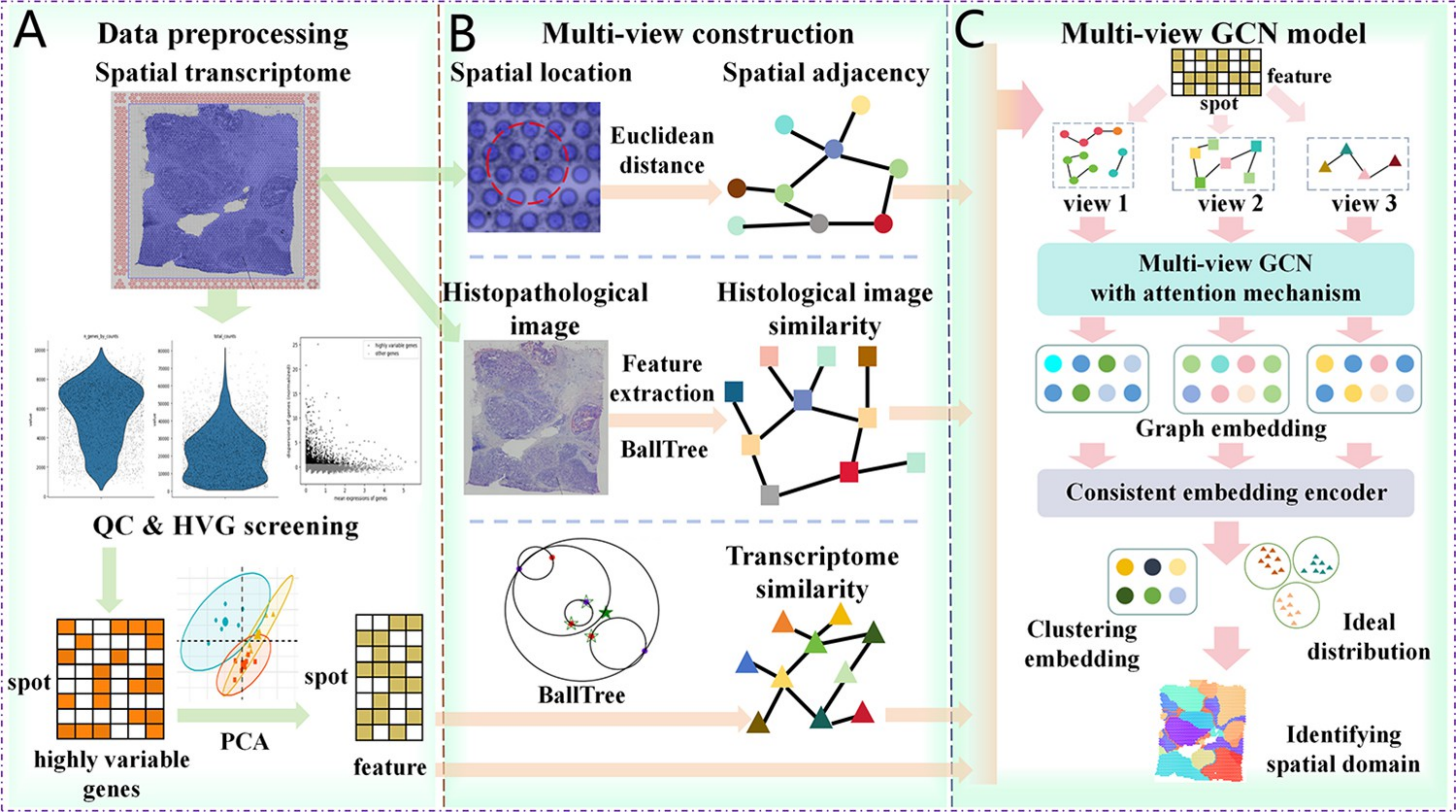
Workflow of MVST. (A) MVST uses SCANPY to perform routine data preprocessing operations such as quality control and highly variable gene screening on spatial transcriptome gene expression data. PCA is used to downscale highly variable gene expression data to obtain a relatively low-dimensional feature representation of spot gene expression data. (B) The graph construction process of MVST reflects its multi-perspective feature, constructing graphs based on distance similarity, histopathological image similarity, and gene expression similarity. The spatial adjacency, histological image similarity, and gene expression similarity networks are obtained through this process. (C) The multi-view graph convolution model of MVST includes a multi-view graph convolution encoder with an attention mechanism and a coherent embedding encoder, in which the multi-view graph convolution encoder learns the spot features from each of the three views, and the coherent embedding encoder integrates the features of the spot in each view to obtain the final clustered embedding.

MVST learns the embedded features of spots from three perspectives–gene expression, spatial location, and histopathological images–and thus identifies spatial domains. First, the spatial transcriptome gene expression data were preprocessed by quality control and normalization to obtain highly variable genes (HVGs) that expressed a high degree of variation among spots, and principal component analysis (PCA) was performed to reduce their dimensionality (Fig 1A). Next, the Euclidean distance between the spots was calculated based on the spatial coordinate position information of the spots to derive a spatial adjacency network. A gene expression similarity network was constructed through the nearest neighbor algorithm based on the gene expression feature information of the spots obtained from the preprocessing, and the histopathology image feature information corresponding to the spots was extracted and the histology image similarity network was constructed using the nearest neighbor algorithm. This enabled the characterization of the interconnections between the spots (Fig 1B). Finally, the gene expression features and the constructed three-view networks were input into a multi-view graph convolutional encoder (the multi-view graph convolutional encoder contains three graph convolutional encoders, each of which corresponds to one view network) with an attention mechanism to learn the low-dimensional graph embeddings of the spots in each view. The resulting graph embeddings were further input into a coherent embedding encoder to obtain the final clustered embeddings and the ideal distributions based on geometric relationships and probability distributions, and then to identify the spatial domains (Fig 1C).

### MVST applied to human breast cancer tissue

**Fig 2:**
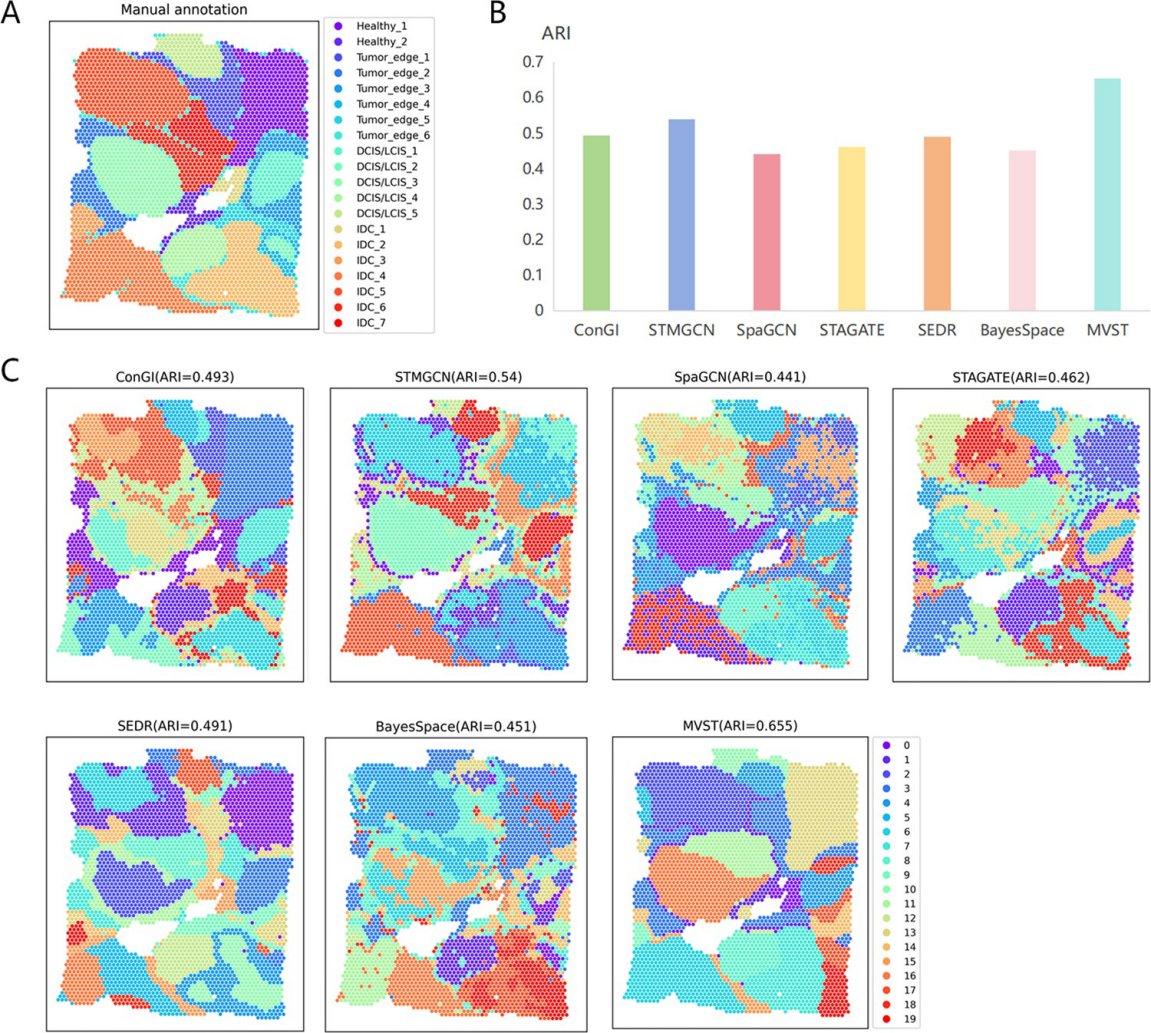
MVST improves spatial domain recognition of human breast cancer tissues. (A) Manual annotation of the spatial domain of the human breast cancer dataset by dividing the tissue slices into 20 regions. (B) Histogram of the performance of spatial domain recognition of human breast cancer dataset using MVST and existing state-of-the-art algorithms (ConGI, STMGCN, SpaGCN, STAGATE, SEDR, and BayesSpace), with the X-axis showing the names of the algorithms and the Y-axis showing the ARI value of the spatial domain recognition results of each algorithm. This is used to compare the results of the predicted spatial domains of each algorithm to the similarity of manually annotated regions. (C) ARI values and visualizations of spatial domain recognition results from MVST and other algorithms for the human breast cancer dataset.

To quantitatively evaluate the performance of MVST for the spatial domain recognition of human cancer tissues, we first applied it to a human breast cancer tissue dataset, a 10X Visium dataset. The human breast cancer tissue dataset, which has been manually annotated, consists of four main morphological types: ductal carcinoma in situ (DCIS/LCIS), healthy tissue, invasive ductal carcinoma (IDC), and tumor margins, and has been further subdivided into 20 regions (Fig 2A). We compared MVST with existing state-of-the-art methods on the benchmark dataset; specifically, MVST was compared with six more advanced spatial clustering methods, namely, ConGI, STMGCN, SpaGCN, STAGATE, SEDR, and BayesSpace, in terms of clustering accuracy in terms of the adjusted RAND index (ARI). The results showed a clear advantage over the other six spatial clustering algorithms, with MVST achieving the best clustering accuracy (ARI = 0.655), and its performance was 11.5% higher in terms of ARI than that of the STMGCN algorithm, which was the second-best performer (Fig 2B and 2C).

By visualizing the spatial domain identification results of MVST with the other five spatial clustering algorithms on the human breast cancer tissue dataset, it is clear that MVST can identify the expected tissue structure more efficiently (Fig 2C). The clustering results of ConGI, STMGCN, SpaGCN, STAGATE, and BayesSpace roughly conformed to the tissue structure of human breast cancer tissue in the major partitions; their clustering boundaries were discontinuous, and there were more anomalies, which affected the accuracy of clustering. The clustering results of the SEDR, although with clearer boundaries, were still far from the actual human breast cancer tissue structure. The clustering results of STMGCN, SpaGCN, STAGATE, and SEDR clearly confused non-tumor regions and some tumor regions, which had a negative impact on the study of tumor tissues in patients with cancer. The clustering results of MVST have clearer boundaries, and at the same time, they are more consistent with the human breast cancer tissue structure partitioning; MVST has a particularly good recognition effect for Healthy_1, and the IDC part of the region is particularly well recognized. These results illustrate the superiority of MVST for identifying the spatial domains of human tumor tissues.

### MVST applied to human dorsolateral prefrontal cortex tissue

Next, we applied MVST to a human dorsolateral prefrontal cortex (DLPFC) tissue dataset, a 10X Visium dataset, to further test the ability of MVST to identify the spatial domains of normal human tissues. The human dorsolateral prefrontal cortex (DLPFC) tissue dataset mentioned above contains 12 tissue section datasets that have been manually annotated based on genetic markers and cellular structures, and the human DLPFC tissue samples are divided into six cortical layers and one white matter layer. We selected slices 151509 and 151510 from this human dorsolateral prefrontal cortex tissue dataset as the benchmark dataset and compared our MVST method with six spatial clustering methods, namely ConGI, STMGCN, SpaGCN, STAGATE, SEDR, and BayesSpace, which are more advanced spatial clustering methods for spatial domain identification, thus verifying the good performance of our method.

**Fig 3:**
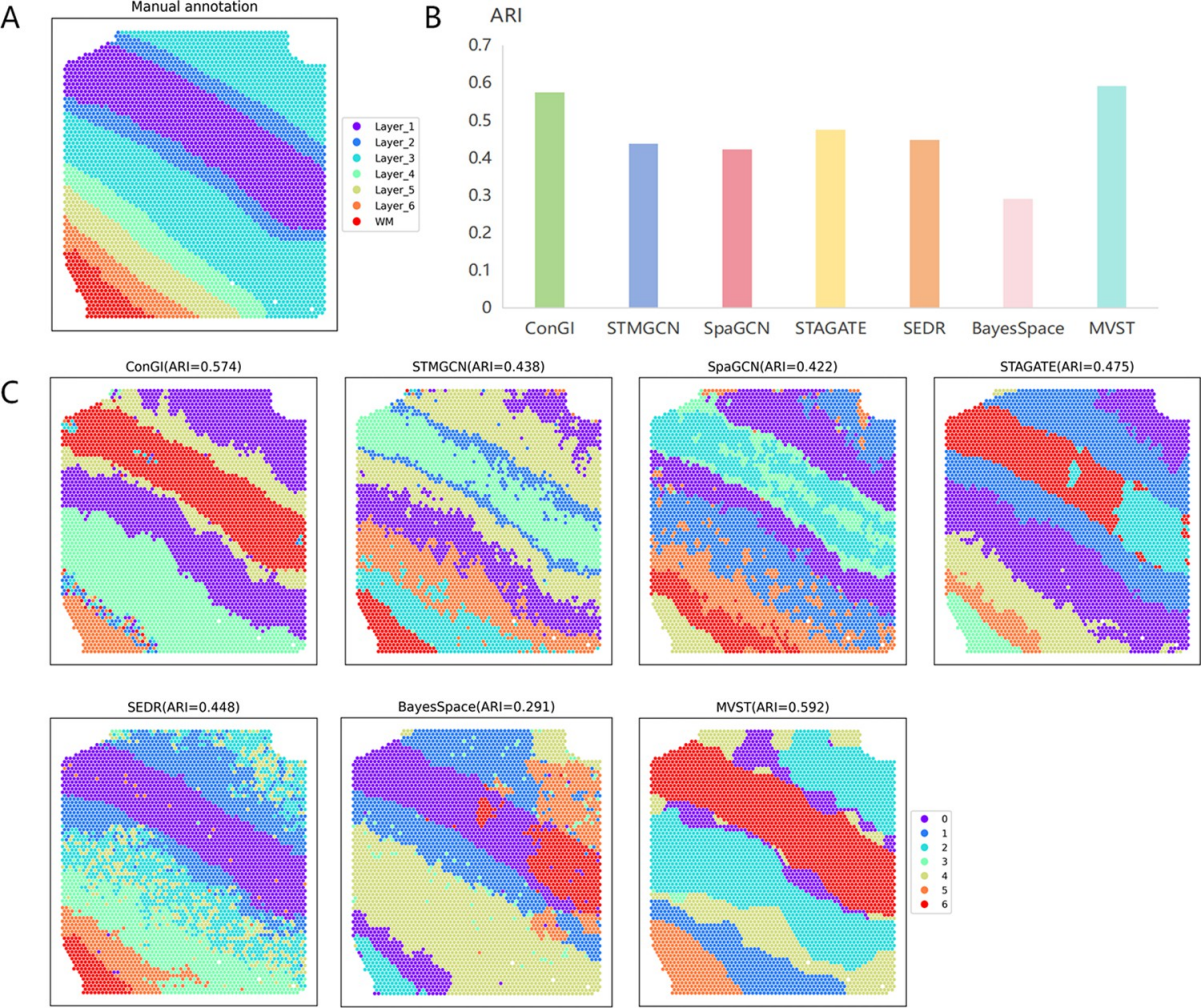
MVST enhances spatial domain recognition of human dorsolateral prefrontal cortex tissue slice 151509. (A) Manual annotation of the spatial domain of human dorsolateral prefrontal cortex tissue slice 151509 dataset by dividing the tissue slice into seven layers. (B) Histogram of the performance of spatial domain recognition of human dorsolateral prefrontal cortex tissue slices 151509 dataset using MVST and existing state-of-the-art algorithms (ConGI, STMGCN, SpaGCN, STAGATE, SEDR, and BayesSpace), with the X-axis showing the names of the algorithms, and the Y-axis showing the ARI of the spatial domain recognition results value of each algorithm, which is used to compare the similarity between the spatial domains predicted by each algorithm and the manually annotated layers. (C) ARI values and visual presentation of the spatial domain recognition results of MVST and other algorithms for the human dorsolateral prefrontal cortex tissue slice 151509 dataset.

The spatial domain division of the manual annotation of human dorsolateral prefrontal cortex tissue slices 151509 is shown in Fig 3A. For the human dorsolateral prefrontal cortex tissue section 151509 dataset, first, with respect to the evaluation criterion of the ARI used, the methods STMGCN, SpaGCN, STAGATE, SEDR, and BayesSpace performed less than optimally, and ConGI was similar to the ARI value of the result of spatial domain identification obtained by our method. However, it was still slightly inferior, approximately 2% lower than the ARI values of the results obtained by our method (Fig 3B and 3C). In contrast, MVST achieved the highest clustering accuracy (ARI = 0.592) for the DLPFC151509 dataset (Fig 3C). Second, from the visualization of the spatial domain recognition results of all the methods used, all these methods roughly identified the hierarchical structure of the human dorsolateral prefrontal cortex tissue slices 151509; however, there were also large differences in the details (Fig 3C). Specifically, the tissue layering identified by BayesSpace was notably different from the true layered structure of human dorsolateral prefrontal cortex tissue section 151509. The three methods, STMGCN, SpaGCN, and SEDR, identified the overall organizational structure of the human dorsolateral prefrontal cortex tissue in the distribution; however, their spatial domain recognition results contained a large number of outliers, which could not correctly distinguish the various layers of tissues, and had a poor grasp of the spatial patterns of the spots. STAGATE’s spatial domain identification results on the DLPFC151509 dataset, although containing only a relatively small number of anomalies, had a large error in the partitioning of each organizational layer, which may have occurred because of an overreliance on spatial relationships between spots during spatial domain identification. Although ConGI identifies the organizational layers Layer_1, Layer_2, Layer_3, and VM more smoothly and clearly, it obfuscates Layer_4, Layer_5, and Layer_6 and identifies numerous anomalies in Layer_6. Our MVST method not only identifies the DLPFC151509 organizational hierarchy as correctly as possible in general, but also the identification of layers Layer_4, Layer_5 and Layer_6 by MVST is significantly more detailed than by ConGI.

**Fig 4:**
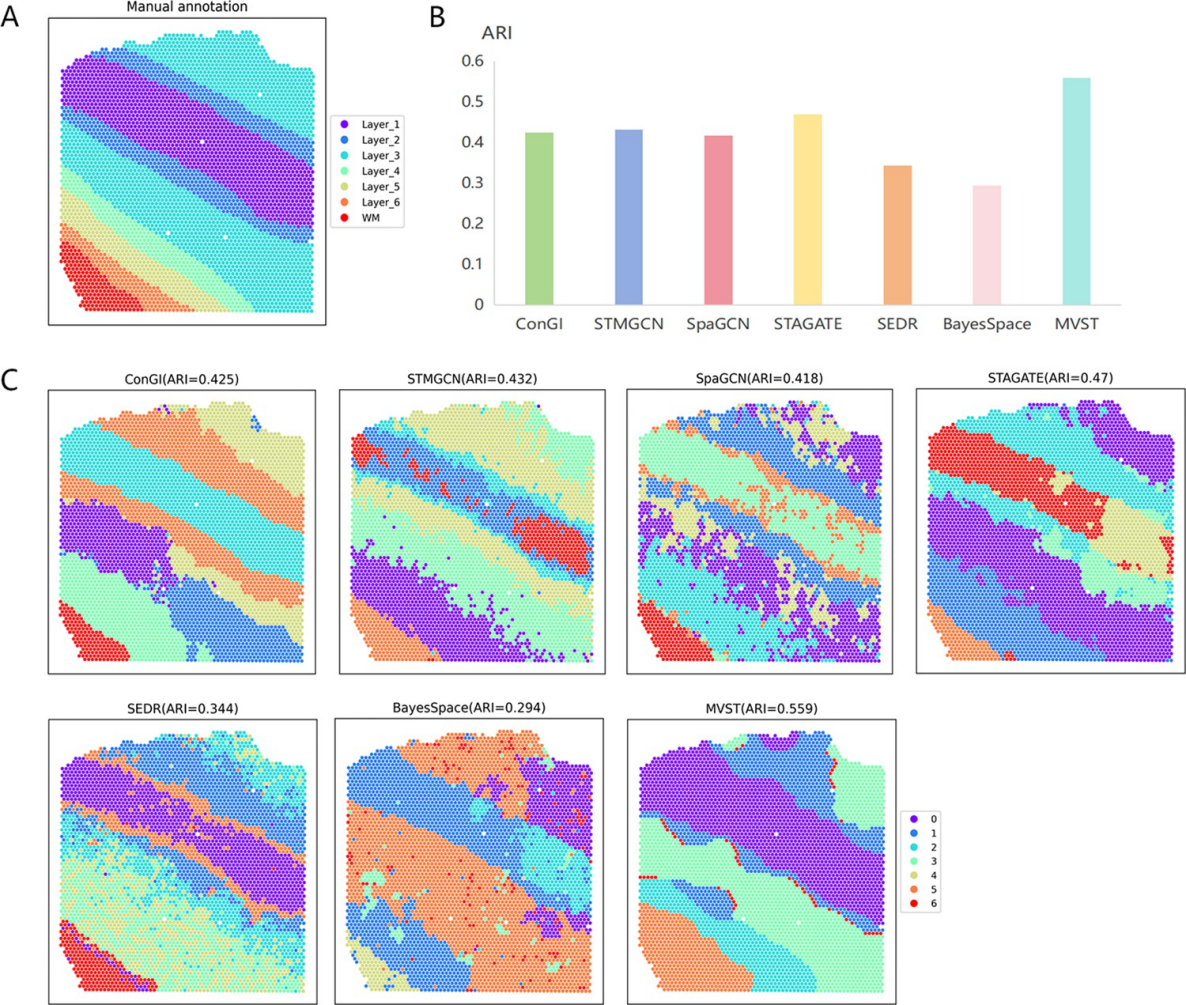
MVST enhances spatial domain recognition of human dorsolateral prefrontal cortex tissue slice 151510. (A) Manual annotation of the spatial domain of human dorsolateral prefrontal cortex tissue slice 151510 dataset by dividing the tissue slice into seven layers. (B) Histogram of the performance of spatial domain recognition of the human dorsolateral prefrontal cortex tissue slices 151510 dataset using MVST and existing state-of-the-art algorithms (ConGI, STMGCN, SpaGCN, STAGATE, SEDR, and BayesSpace), with the X-axis showing the name of each algorithm, and the Y-axis showing the ARI of the spatial domain recognition results of each algorithm value, which is used to compare the similarity between the spatial domains predicted by each algorithm and the manually annotated layers. (C) ARI values and visual presentation of spatial domain recognition results from MVST and other algorithms for the human dorsolateral prefrontal cortex tissue slice 151510 dataset.

Next, the spatial domains of another tissue slice of the human dorsolateral prefrontal cortex, slice 151510, were identified using our MVST method, along with the six methods mentioned above, which are currently more advanced. Manually annotated spatial domain delineation of the human dorsolateral prefrontal cortex tissue slice 151510 is shown in Fig 4A, which is similar to the delineation of the DLPFC151509 slices. A plot of the spatial domain recognition results and a comparison of the clustering accuracy of each method for the human dorsolateral prefrontal cortex dataset slice 151510 are shown in Fig 4B and 4C, respectively. It can be seen that MVST is closest to the manually annotated stratification in terms of the ARI value, its spatial domain recognition results have the highest clustering accuracy compared to that of the other method (ARI = 0.559), and it outperforms the second-best-performing ConGI method by approximately 13%, which is a clear advantage. Second, in terms of the visualization of the spatial domain identification results, the spatial domains identified by MVST were relatively more consistent with manual annotation stratification. Although the boundaries of the spatial domains identified by ConGI and STAGATE were more distinct, some layers of the identified spatial domains were divided into two halves, which is inconsistent with the general rule of manual annotation layering. The spatial domains identified by the three methods, STMGCN, SpaGCN, and SEDR, were similar to their performances on DLPFC151509, with point-level noise, showing obvious fragmentation characteristics. The spatial domain identified by BayesSpace was similar to its performance on DLPFC151509, with the identified organizational hierarchies differing from the true hierarchical structure in a nontrivial manner.

From the above spatial domain recognition results and their analyses, compared with the six currently more advanced spatial domain recognition methods, namely ConGI, STMGCN, SpaGCN, STAGATE, SEDR, and BayesSpace, MVST has a stronger spatial domain recognition capability for human dorsolateral prefrontal cortex tissues, and the identified spatial domains are more in line with the true stratification of the tissues.

### MVST applied to mouse anterior brain tissue

**Fig 5:**
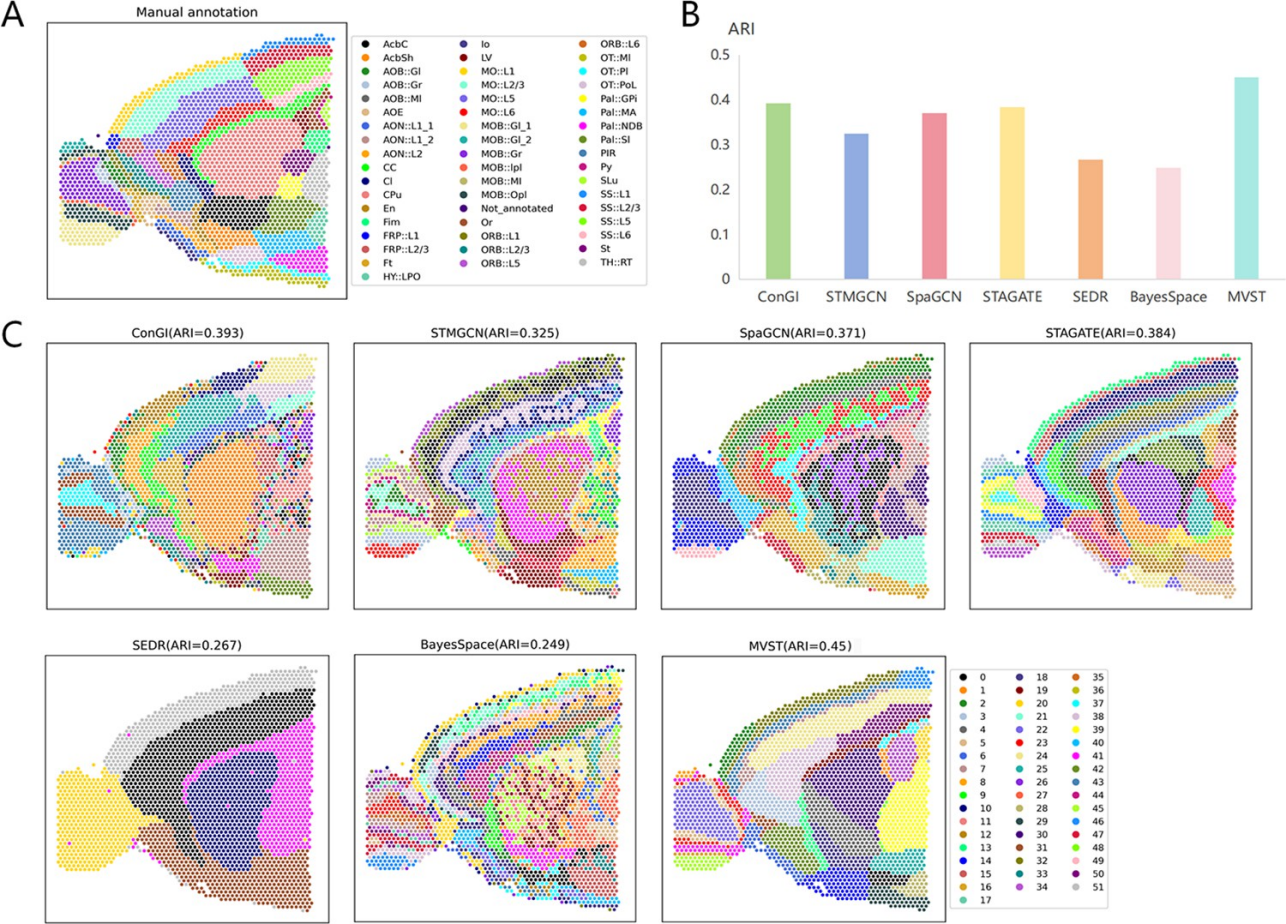
MVST improves the spatial domain recognition of mouse anterior brain tissue slices. (A) Manual annotation of the spatial domains of the mouse anterior brain tissue slice dataset by dividing the tissue slices into 52 regions. (B) Histogram of the performance of spatial domain recognition of the mouse anterior brain tissue slices dataset using MVST and existing state-of-the-art algorithms (ConGI, STMGCN, SpaGCN, STAGATE, SEDR, and BayesSpace), with the X-axis showing the names of the algorithms, and the Y-axis showing the ARI value of the spatial domain recognition results of each algorithm, which is used to compare the similarity between the spatial domains predicted by each algorithm and the manually annotated layers. (C) ARI values and visual presentation of spatial domain recognition results of MVST and other algorithms on the mouse anterior brain tissue slice dataset.

In addition to applying MVST to a spatial transcriptome dataset of human tissues, we also applied MVST to a dataset of mouse anterior brain tissue slices to further test the spatial domain recognition capability of our method in tissues of species other than humans. The slices of the mouse anterior brain tissue slice dataset that we used were manually annotated, and they were more meticulously divided into 52 spatial domains (Fig 5A), which posed a considerable challenge to the spatial domain recognition algorithm. To evaluate the performance of MVST for spatial domain recognition on mouse anterior brain tissue slices, we compared MVST with the existing state-of-the-art methods mentioned above on the above benchmark dataset, and the results showed that our MVST method also achieved the best spatial domain recognition results in comparison with the mouse anterior brain tissue slice dataset. The clustering precision of the spatial domain recognition results, ARI, was up to 0.45, which is more than 5% higher than the ARI value obtained by the next best performing method, ConGI (Fig 5B and 5C). In terms of the delineation of the spatial domain, MVST has a better grasp of the general structure of the mouse anterior brain tissue slices; in particular, it better identifies the MOB region of the mouse anterior brain tissue. The boundaries of the spatial domain identification results of MVST are smoother, and at the same time there is almost no point-level noise. For the mouse anterior brain tissue slice dataset, SEDR was unable to identify the spatial domains in detail, and only a few rough delineations of the tissue slices were performed. Although ConGI, STMGCN, SpaGCN, and BayesSpace can perform a more detailed delineation of the spatial domains, the spatial domains identified by these methods appear slightly chaotic and more fragmented. The spatial domains delineated by STAGATE are more detailed, and the delineation boundaries are smoother; however, their similarity to the real configuration of the tissue in the anterior part of the mouse brain is lower, which can also be seen in the ARI values. In summary, MVST can achieve good results in the spatial domain recognition of mouse tissues and can also perform more detailed spatial domain recognition of tissues with complex structures.

### MVST applied to simulated data

**Fig 6:**
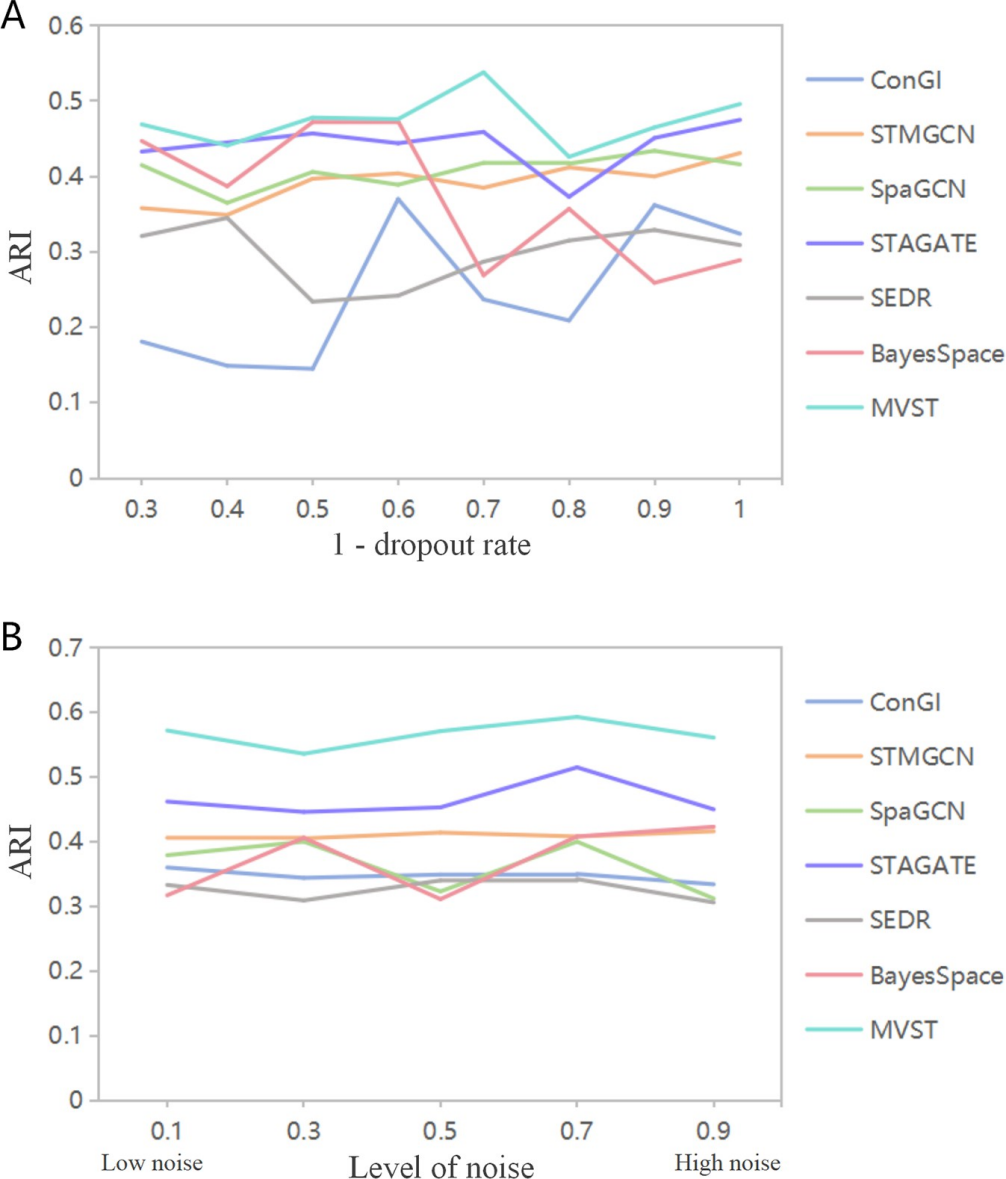
Performance comparison between MVST and six comparison methods on simulated data. (A) Performance comparison between MVST and six comparison methods when facing different sparsities of gene expression matrix; the X-axis represents different sparsities of gene expression matrix and the Y-axis represents ARI values. (B) Performance comparison between MVST and the six comparative methods when facing simulated data with different levels of added noise. The X-axis represents the level of added noise and the Y-axis represents the ARI value.

As a result of experiments on simulated datasets that may better reflect the wide applicability and robustness of spatial domain recognition methods, we applied and compared the performance of MVST and six more advanced spatial domain recognition methods, such as ConGI, to simulated datasets constructed based on the human dorsolateral prefrontal cortex tissue slice 151509 dataset to further validate the robustness of MVST. The experimental results showed that the spatial domain recognition effect of ConGI and BayesSpace fluctuated greatly in the face of changes in gene expression matrix sparsity, whereas the performance of STMGCN, SpaGCN, STAGATE, SEDR, and MVST was less affected by changes in gene expression matrix sparsity, and its performance was relatively stable (Fig 6A). Moreover, from the ARI values, the spatial domain recognition accuracy of our MVST method was mostly higher than that of the other methods when facing gene expression matrices with different sparsities. When facing simulated data with different levels of noise added, MVST and the remaining six spatial domain recognition methods show good robustness, and, from the ARI values, the spatial domain recognition accuracies of our method MVST are always higher than those of the other compared methods (Fig 6B). In summary, we conclude that the MVST is highly robust.

### Ablation study

**Fig 7:**
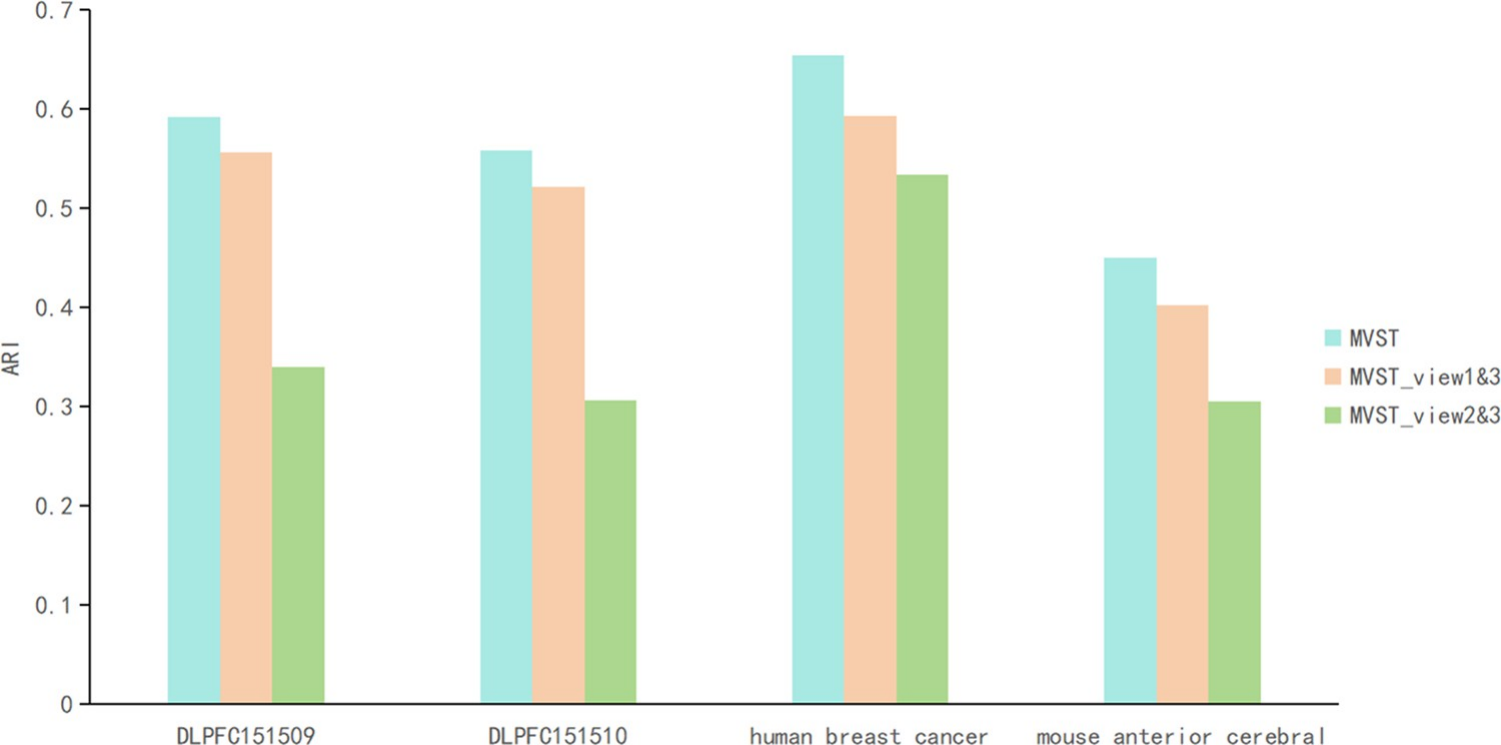
Comparison of the performance of MVST with its two variants (MVST_view1&3 and MVST_view2&3) on the human dorsolateral prefrontal cortex, mouse anterior cerebral slice, and human breast cancer datasets, with the X-axis representing the different datasets and the Y-axis representing the ARI values.

In spatial transcriptomics, it is often assumed that cells adjacent to each other at spatial locations will exhibit similar gene expression profiles. The architecture of MVST uses both spatial neighborhood network maps and gene expression similarity network maps, which implies that MVST may capture redundant information, so we conducted ablation experiments by designing two variants of MVST to explore whether the possible capture of redundant information would have an impact on the spatial domain recognition effect of MVST. The first MVST variant uses only two views of the spatial adjacency network graph and histological image similarity network graph for spatial domain identification and is named MVST_view1&3. The second MVST variant uses only two views of the gene expression similarity network graph and the histological image similarity network graph for spatial domain identification and is named MVST_view2&3. We applied MVST and its two variants to the human dorsolateral prefrontal cortex dataset, mouse anterior brain slice dataset, and human breast cancer dataset used in the above ablation experiments. The results of the ablation experiments showed that the spatial domain recognition of MVST was better than that of the two variants of MVST on all three real spatial transcriptome datasets, including the human dorsolateral prefrontal cortex dataset, demonstrating that the framework structure of MVST is necessary and more reasonable (Fig 7). In addition, the results of the ablation experiments show that spatial and gene expression information contain cell grouping information that may not be identical; these two can complement each other in MVST to achieve better results, and there is no degradation of the spatial domain recognition performance owing to the possible redundancy of information. In the ablation experiments, MVST_view1&3 outperformed MVST_view2&3, suggesting that spatial location neighbourhood information may be more important than gene expression similarity information. This phenomenon corresponds to the fact that spatial information is crucial for spatial domain identification as described in the SpaGCN correspondence literature and others.

## Discussion

To fully integrate the spatial information, gene expression information, and histopathological image information of spatial transcriptomic data to explore the structure of organ tissues, we proposed MVST in this study. This process captures the similarity between spots in terms of gene expression, spatial location, and histopathological images from spatial transcriptomic data separately and learns the embedding of each spot using a multiview map convolutional encoder with consistency encoder representation. MVST integrates histopathological images, gene expression, and spatial location information into spatial transcriptomic data, thus identifying spatial domains from a more comprehensive perspective. To validate the performance of our method, we tested MVST on three real spatial transcriptomics datasets. Our experimental results show that the MVST algorithm has a better competitive performance than existing methods.

In terms of processing histopathological images, MVST is different from existing methods such as SpaGCN and stLearn [37], which take histological images as input. It uses the features of this view of histopathological images to construct the graph structure individually and obtain embeddings in the graph convolutional encoder, which are then integrated with the embeddings obtained through the gene expression information view and the spatial location information view, respectively, to obtain the final clustering embedding. Methods such as SpaGCN and stLearn are more inclined to incorporate the features of histopathology images into the gene expression information and spatial location information of the spot, which are then uniformly fed into the algorithms or models for processing. Meanwhile, the model of MVST is flexible, allowing the user to adjust the weighting of three views: gene expression information, spatial location information, and histopathological image information. Moreover, based on the experiments we have conducted, the boundaries of the spatial domains identified by MVST on the datasets we used are relatively smooth, with no fragmented spatial domains. We hypothesize that this feature of MVST is more in line with exploring the organizational structure.

Irrespective of its good performance in spatial domain recognition, MVST has some limitations. One limitation of MVST is that it mainly supports spatial domain recognition on spatial transcriptome datasets generated by the currently dominant spatial transcriptome sequencing technology, 10X Visium. Therefore, the applicability of the method has to be further improved in future work, and we expect that the design idea of the MVST method can be extended to spatial transcriptome data with higher resolution in the near future. Another limitation is that the extraction, integration, and exploitation of histopathological image features of MVST are based on simpler ideas; therefore, the processing of histopathological images needs to be optimized as much as possible to make full use of these features in the future. In addition, the measure of similarity between spots of MVST in terms of gene expression and histopathological images is based on the BallTree algorithm, an optimized algorithm for KNN. Better results may be achieved if a more biologically meaningful approach is used in the next step. We expect that research in these areas will further improve the performance of MVST. From the perspective of research in the field of spatial domain recognition, with an increase in spatial transcriptome technology and the size of spatial transcriptome data, it is necessary to improve the efficiency and scalability of spatial domain recognition tools. Moreover, at present, large-language models such as BERT have achieved good results in the field of bioinformatics, and if large-language models are applied to spatial domain recognition, they may also achieve good results and breakthroughs.

## Materials and methods

### Data description

We applied MVST to three ST datasets generated by the currently dominant spatial transcriptome sequencing technology, 10X Visium, including the human dorsolateral prefrontal cortex [38], mouse anterior cerebral section, and human breast cancer datasets. The human dorsolateral prefrontal cortex dataset contained 12 samples of dorsolateral prefrontal cortex slices provided by the three subjects. The sequenced tissue comprised six neuronal layers and white matter, with a different number of spots for each sample. The mouse anterior brain slice dataset contains 2695 spots, and the slices were manually annotated into 52 regions. The human breast cancer dataset contains 3798 spots, with 24,923 genes sequenced, and the slices were manually annotated into 20 regions. We applied MVST to simulated datasets constructed from the 151509 dataset of human dorsolateral prefrontal cortex tissue slices. Firstly, in terms of different expression matrix sparsities, we constructed simulated data with different sparsities by randomly deleting the count values of gene expression matrices of human dorsolateral prefrontal cortex tissue section 151509 according to different ratios (0, 0.1, 02, 0.3, 0.4, 0.5, 0.6, and 0.7), and thus investigated the impact of gene expression matrix sparsity on our spatial domain recognition tool. When the ratio is 0.7, the probability that each value in the gene expression matrix is set to 0 is 0.7, and conversely, a ratio of 0 implies that the gene expression matrix remains unchanged. It should be noted that when the set ratio is larger than 0.7, the gene expression matrix is too sparse and the dimensions of the features of the spot cannot meet the requirements of the MVST model structure, so the maximum ratio is set to 0.7. Secondly, in terms of adding noise to the dataset, we used a Poisson distribution to add different levels of noise (10%, 30%, 50%, 70% and 90%) to the gene expression matrix of human dorsolateral prefrontal cortex tissue section 151509 to construct the simulated data with different levels of noise added to it, and thus to investigate the effect of different noise levels on our spatial domain recognition tool. Specifically, we used a Poisson distribution with the mean parameter set to the product of the noise level and the mean gene expression level.

### Data preprocessing

For all datasets, the SCANPY [39] tool was used for preprocessing. First, quality control filtering was performed on the gene expression data to filter genes expressed in fewer than five cells. Second, the gene expression data were normalized and legitimized. The top 2000 highly variable genes were then filtered, and the data were scaled. Finally, the preprocessed gene expression data were downscaled using PCA, and the first 1000 principal components were used as feature inputs for the spot. For datasets in which the number of spatial domain divisions is unknown, we use the Louvain algorithm implemented in SCANPY to select the number of spatial domain divisions during the data preprocessing stage.

### Construction of graphs

MVST identifies spatial domains from three perspectives. Therefore, we construct three different undirected graphs as inputs from each of the three perspectives: spatial adjacency network graphs, gene expression similarity network graphs, and histological image similarity network graphs. The details of the three undirected graphs are described below.

#### Spatial adjacency network diagram

To obtain the spatial adjacency between spots in the spatial transcriptome data, we calculated the Euclidean distance between the coordinates of the corresponding spatial locations of any two spots, selected the top 10 nearest spots as spatial neighbors according to the size of the distances between a spot and all other spots, and constructed the adjacency matrix, denoted as *A*. In adjacency matrix *A*, if spots *i* and j have a spatial neighborhood, then the matrix element *a*_*ij*_ = *a*_*ji*_ = 1; otherwise, *a*_*ij*_ = *a*_*ji*_ = 0. At the time of data input, a spatial neighborhood network graph was constructed based on the adjacency matrix *A*.

#### Gene expression similarity network diagram

To obtain the gene expression similarity relationship between spots in the spatial transcriptome data, the first two thousand preprocessed high-variance gene expression data points were first downscaled to 50 dimensions using PCA, and then the Ball Tree algorithm [40] was used to obtain the top 10 nearest neighbors of each spot in terms of gene expression similarity based on the downscaled gene expression features of the spot and construct the neighbor-joining matrix, denoted as *B*. In neighbor-joining matrix *B*, the matrix element *b*_*ij*_ = *b*_*ji*_ = 1 if spot *i* has a gene expression similarity relationship with spot *j* otherwise, *b*_*ij*_ = *b*_*ji*_ = 0. At the time of data input, a gene expression similarity network graph was constructed based on the neighbor-joining matrix *B*.

#### Histological image similarity network diagram

To obtain the histological image similarity relationship between spots in spatial transcriptome data, first, the image features at the corresponding position of each spot were extracted from the H&E-stained histopathological images; then, the image features of the spots were normalized using the RobustScaler normalization method; finally, the Ball Tree algorithm was used to obtain the top 10 nearest neighbors for each spot’s top 10 nearest neighbors in terms of histological image similarity and construct the adjacency matrix, denoted as *C*. In the adjacency matrix *C*, if spot *i* has a histological image similarity relationship with spot *j*, then the matrix element *c*_*ij*_ = *c*_*ji*_ = 1; otherwise, *c*_*ij*_ = *c*_*ji*_ = 0. At the time of data input, the histological image similarity network graph was constructed based on adjacency matrix *C*.

Histopathology image feature extraction specifically involves extracting the texture and intensity features of the image corresponding to the position of each spot in the histopathology image through local (adaptive) histogram equalization and computation of gray-scale covariance matrices. Among them, the image texture feature is a global feature that can reflect important texture patterns and structural information in the image. We quantitatively describe the texture feature by calculating contrast, non-similarity, homogeneity, angular second-order moment (ASM), entropy, and correlation. The RGB three-channel intensity feature of the image corresponding to the position of each spot in the histopathological image was extracted as the intensity feature of the spot.

### MVST Architecture

MVST is based on graph neural networks to learn the embedding features of three views–spatial neighborhood network graph, gene expression similar network graph, and histological image similar network graph–and to learn the view consistency embedding. Specifically, the MVST has two modules: a multiview graph convolutional encoder and a consistent embedding encoder. The first module, the multi-view graph convolutional encoder, was used to learn the graph embedding features of the spot in the three views to reduce noise and redundancy. The second module, a Consistent Embedding Encoder, captures the consistency of the geometric relationships and probability distributions among different views to learn consistent cluster embeddings for spatial domain recognition.

#### Multi-view graph convolutional encoder

In a multi-view graph convolutional encoder, the *m*-*th* view maps the spot feature and the *m*-*th* view *G*_*m*_ to the *d*-dimensional graph embedding feature *H*_*m*_ and the output of the *l-th* multi-view graph convolutional encoder is shown by the following equation:

$$H_{m}^{\left(l\right)}=\sigma (D^{-\frac{1}{2}}G_{m}\prime D^{-\frac{1}{2}}H_{m}^{\left(l-1\right)}W^{\left(l\right)})$$ (1)

where *G*_*m*_*’ = G*_*m*_ *+ IN* is the relevance coefficient matrix with added self-connection, *IN* is the identity matrix, *D*_*ii*_ = ∑_*j*_*G*_*m*_′_*ij*_, *W*^*(l)*^ is the trainable parameter of the *l-th* multi-view auto-encoder layer and *σ* denotes the activation function.

After applying *L* multiview encoder layers, we obtain the graph embedding *H*_*m*_, which retains all the information regarding the multiview graph structure *G* and node feature matrix *X*. Then, we decode the feature *H*_*m*_ containing almost all the information based on different views. In this process, we used the same number of layers as the decoders, and each decoder layer tries to invert its corresponding encoder layer. In other words, the decoding process is the inverse of the encoding process.

In order to determine the correlation between nodes and neighboring points, we use an attention mechanism that shares parameters between nodes. The learnable correlation matrix *U* in each view is defined by the following equation:

$$U=\varphi (G_{m}⊙t_{s}^{\left(l\right)}H_{m}^{\left(l\right)}W^{\left(l\right)}+G_{m}⊙t_{r}^{\left(l\right)}H_{m}^{\left(l\right)}W^{\left(l\right)})$$ (2)

where $t_{s}^{\left(l\right)}$ and $t_{r}^{\left(l\right)}\in R^{1\times d_{l}}$ represents the trainable parameters related to their own nodes and neighbor nodes, respectively; ⊙ refers to the element-wise multiplication with broadcasting capability; and *φ* denotes the activation function which is generally set as the sigmoid activation function.

The normalization matrix *U* was passed through to obtain the final correlation coefficient *Y*. Thus, *Y*_*ij*_ was calculated using the following equation:

$$Y_{ij}=\frac{exp(U_{ij})}{\sum_{k\in N_{i}}exp(U_{ik})}$$ (3)

where *N*_*i*_ is the set of all nodes adjacent to node *i*.

#### Consistency embedded encoder

In the consistent embedding encoder, for view *m*, we first employ a nonlinear feature extraction mapping embedding *H*_*m*_, which is mapped to the low-dimensional space *Z*_*m*_. We then learn a common clustering embedding *Z* that integrates all *Z*_*m*_, *Z* can be computed using the following equation:

$$Z=\beta_{1}Z_{1}+\beta_{2}Z_{2}+\beta_{3}Z_{3}$$ (4)

where *β*_*1*_, *β*_*2*_, *β*_*3*_ are hyperparameters and *β*_*1*_ *+ β*_*2*_ *+ β*_*3*_ *= 1*.

We use the Student’s t-distribution, which measures the similarity between the integrated node representation *z*_*i*_ and the center of mass *μ*_*j*_. Thus, the original probability distribution *Q* of *Z* is shown by this equation:

$$q_{ij}=\frac{{\left(1+{\left||z_{i}-\mu_{j}|\right|}^{2}/\alpha \right)}^{-\frac{\alpha +1}{2}}}{\sum_{j\prime }{\left(1+{\left||z_{i}-\mu_{j\prime }|\right|}^{2}/\alpha \right)}^{-\frac{\alpha +1}{2}}}$$ (5)

where ${\left\{\mu_{j}\right\}}_{j=1}^{k}$ is the *k* initial cluster centroids, *α* is the degree of freedom of the Student’s t-distribution, *q*_*ij*_ is the probability of assigning node *i* to cluster *j*.

Next we compute the target probability distribution P of *Z*. *p*_*ij*_ is an element of P with *0 ≤ p*_*ij*_ *≤ 1*. As shown in the equations below, we obtain *p*_*ij*_:

$$p_{ij}=\frac{q_{ij}^{2}/f_{i}}{\sum_{j\prime }q_{ij\prime }^{2}/f_{j\prime }}$$ (6)

where *f*_*i*_ *=* ∑_*i*_*q*_*ij*_ are soft cluster frequencies.

#### Loss function

The loss function consists of the reconstructed multiview node feature matrix loss *L*_*e*_, geometric relationship consistency loss *L*_*gr*_, and probability distribution consistency loss *L*_*pd*_, which can be calculated using the following equations:

$$L_{e}=\underset{\theta }{\min}\sum_{i=1}^{M}{\left||X-{X\prime }_{i}|\right|}_{F}^{2}$$ (7)

$$L_{gr}=\underset{\gamma }{\min}\sum_{i\neq j}^{M}{\left||Z_{i}-Z_{j}|\right|}_{F}^{2}$$ (8)

$$L_{pd}=\underset{\gamma }{\min}\sum_{m=1}^{M}{\rho_{m}||Q_{m}-P||}_{F}^{2}$$ (9)

$$L=L_{e}+L_{gr}+L_{pd}$$ (10)

where *M* is the number of views (3 in the experiment), *θ* and *γ* are the network parameter of the multi-view map convolutional encoder, *Q*_*m*_ is the soft distribution of *Z*_*m*_, *P* is the auxiliary distribution of *Z*, and *ρ* is the trade-off parameter.

#### Spatial domain identification through clustering

First, an auxiliary distribution P is learned from the clustered embedded features *Z* and then, the category label of each node is predicted from the auxiliary distribution P. For node *i*, the index with the highest probability value in *p*_*i*_ is its category label. The specific calculation process equation is as follows.


$$y_{i}=\underset{k}{\mathrm{argmax}}\left(p_{ik}\right)$$ (11)

### Evaluation criteria and baseline methodology

#### Evaluation criteria

We used the ARI [41], a commonly used clustering index, as an evaluation criterion for the clustering results. The equation for ARI is as follows:

ARI=∑i∑j(nij2)−∑i(ai2)∑j(bj2)(n2)∑i(ai2)+∑j(bj2)2−∑i(ai2)∑j(bj2)(n2) (12)

where *n*_*ij*_ is the number of data points within the same category *u*_*i*_ and cluster *v*_*j*_, *a*_*i*_ is the number of data points in category *u*_*i*_; *b*_*j*_ is the number of data points in cluster *v*_*j*_; *n* is the total number of data points.

#### Baseline methodology

To evaluate the performance of our method in identifying spatial domains, we compared MVST with ConGI, STMGCN, SpaGCN, STAGATE, SEDR, and BayesSpace. For all compared methods, we tested them on all datasets using default parameters and default data preprocessing processes and used the same number of real clusters in the clustering.
